# Triglyceride-glucose body mass index predicts prognosis in patients with ST-elevation myocardial infarction

**DOI:** 10.1038/s41598-023-51136-7

**Published:** 2024-01-10

**Authors:** Ming Liu, Jianyuan Pan, Ke Meng, Yuwei Wang, Xueqing Sun, Likun Ma, Xiaofan Yu

**Affiliations:** 1 Department of Cardiology, The First Affiliated Hospital of USTC, Division of Life Sciences and Medicine, University of Science and Technology of China, Hefei, 230001 Anhui China; 2 Department of Cardiology, Anhui Provincial Hospital Affiliated of Anhui Medical University, Hefei, 230001 Anhui China; 3 The Second Hospital of Jilin University, Changchun, 130000 Jilin China

**Keywords:** Prognostic markers, Endocrine system and metabolic diseases

## Abstract

Triglyceride glycemic-body mass index (TyG-BMI) is a simple and reliable surrogate for insulin resistance (IR). However, it is still unclear if TyG-BMI has any predictive value in patients having percutaneous coronary intervention (PCI) for ST-segment elevation myocardial infarction (STEMI). The purpose of this study was to examine the TyG-BMI index's prognostic significance and predictive power in patients with STEMI. The study comprised a total of 2648 consecutive STEMI patients who underwent PCI. The primary endpoint was the occurrence of major adverse cardiovascular events (MACE), defined as the combination of all-cause death, nonfatal myocardial infarction, nonfatal stroke, and coronary revascularization. The TyG-BMI index was formulated as ln [fasting triglycerides (mg/dL) × fasting plasma glucose (mg/dL)/2] × BMI. 193 patients in all experienced MACE over a median follow-up of 14.7 months. There was a statistically significant difference between the Kaplan–Meier survival curves for the TyG-BMI index tertiles (log-rank test, *p* = 0.019) for the cumulative incidence of MACE. The adjusted HRs for the incidence of MACE in the middle and highest quartiles of the TyG-BMI index compared with the lowest quartile were 1.37 (95% CI 0.92, 2.03) and 1.53 (95% CI 1.02, 2.29), respectively, in the fully adjusted Cox regression model. At six months, one year, and three years, the TyG-BMI area under the curve (AUC) for predicting MACE was 0.691, 0.666, and 0.637, respectively. Additionally, adding the TyG-BMI index to the risk prediction model enhanced outcome prediction. In STEMI patients undergoing PCI, TyG-BMI was independently linked to MACE. TyG-BMI could be a simple and solid way to assess MACE risk and prognosis.

## Introduction

The most serious subtype of coronary artery disease and a significant factor in mortality in the world's population is ST-segment elevation myocardial infarction (STEMI)^1^. The most successful treatment for STEMI now is the quick restoration of coronary blood flow, mostly with percutaneous coronary intervention (PCI)^2^. Over the past several decades, there has been a tremendous improvement in the management of STEMI, along with a significant decrease in mortality^3,4^. Nevertheless, despite direct percutaneous coronary intervention, individuals who survive the STEMI acute phase may continue to experience major adverse cardiovascular events (MACE)^5^. Therefore, it is crucial for improving risk classification in interventional patients, which is a significant issue^6^.

Insulin resistance (IR), which is defined as reduced or impaired insulin sensitivity in insulin-dependent tissues or organs shown by impaired glucose uptake and oxidation^7,8^, is a significant risk factor for the development of type 2 diabetes and coronary artery disease.

The hyperinsulinemic-euglycemic clamp is thought to be the most accurate way to measure IR^9^, but its invasiveness, high cost, and complexity restrict its practical use^10^. Although the dynamic homeostasis model assessment of insulin resistance (HOMA-IR) has gained popularity and recognition as a valid tool, it is not recommended for use in patients who are on insulin therapy^11,12^. The triglyceride glucose (TyG) index and the triglyceride glucose body mass index (TyG-BMI) are two novel alternative indices that may be more practical and viable than existing approaches to predicting the presence of insulin resistance^13,14^. Both the HOMA-IR and the hyperinsulinemic-euglycemic clamp have been shown to have a significant connection with the TyG index^15^. In evaluating IR for the general population, HOMA-IR is inferior to the TyG index^16^. The TyG index is associated with endothelial dysfunction, thrombosis, arterial stiffness, coronary artery calcification, and atherosclerotic disease, according to several recent studies^17,18^.

The TyG-BMI index, which combines obesity measurements with the TyG index, can enhance the capacity to detect IR^19^. Recent research has also revealed that the TyG-BMI index is an independent risk factor for multivessel coronary atherosclerotic heart disease (CAD) in addition to being highly linked with CAD severity^20^. In individuals with coronary heart disease, The TyG-BMI index could be linked to cardiovascular incidents^21^. The effect of the TyG-BMI index on the prognosis of patients with STEMI has, however, received less attention in research. Therefore, this study sought to ascertain if the TyG-BMI index enhanced risk stratification and to investigate the effect of baseline TyG-BMI on the prognosis in STEMI patients having percutaneous coronary intervention.

## Methods

### Study design and population

**Figure 1:**
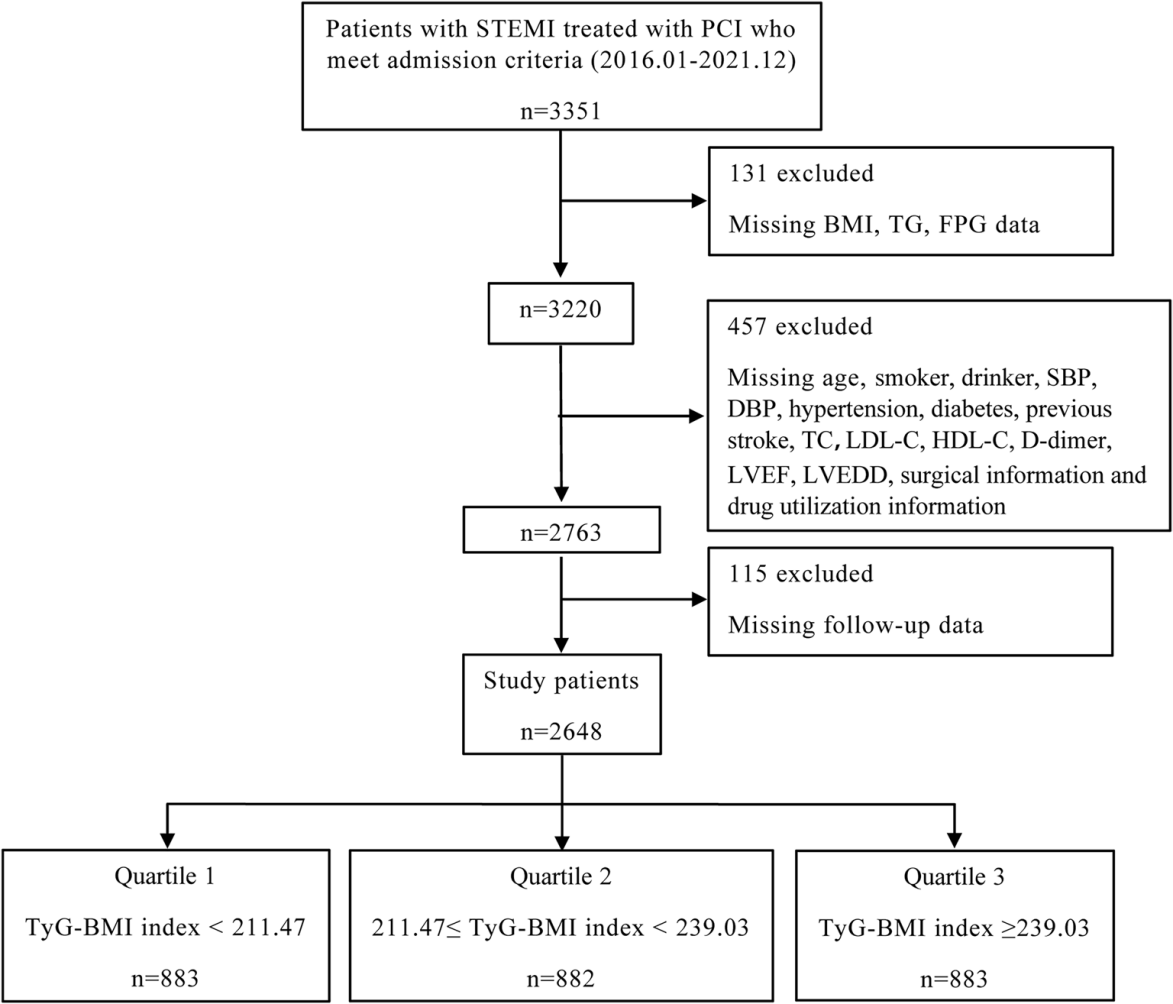
Flowchart of selecting patients for inclusion in the study.

This study was an observational, retrospective cohort study conducted at a single site. Patients with STEMI who received direct PCI at the First Hospital Affiliated with the University of Science and Technology of China (Anhui Provincial Hospital) between January 2016 and December 2021 were included in this study. Clinical evidence of myocardial ischemia and the presence of ST-segment elevation on the electrocardiogram, which is indicative of an acute myocardial infarction, were used to make the STEMI diagnosis^22^. Exclusion criteria included severe valvular heart disease, malignant neoplasms, decompensated heart failure, hematologic diseases, non-ischemic dilated cardiomyopathy, severe hepatic or renal illness, autoimmune disorders, or missing medical data. Percutaneous coronary intervention and coronary angiography were carried out according to industry standards and recommendations^23^. After percutaneous coronary intervention, all patients received conventional dual antiplatelet medication (aspirin 100 mg/day and clopidogrel 75 mg/day) for at least a year. After removing data with incomplete clinical information and patients who were lost to follow-up, a total of 2648 patients were included in this investigation, as shown in Fig. 1.

### Ethics statement

The study complied with the Declaration of Helsinki and was approved by the Ethics Review Committee of the First Hospital Affiliated to the University of Science and Technology of China (Anhui Provincial Hospital). Furthermore, written informed consent was obtained from all patients.

### Data collection and definitions

At the time of admission, we gathered from patients general clinical data (age, sex, height, and weight), medical history data (previous acute myocardial infarction, previous stroke, previous PCI, previous arrhythmia, family history of coronary artery disease, hypertension, diabetes mellitus, smoking, and alcohol consumption), and diagnostic data. Following a 12-h fast, patients were subjected to normal laboratory testing for blood sugar, creatinine, blood urea nitrogen (BUN), high-density lipoprotein cholesterol (HDL-C), low-density lipoprotein cholesterol (LDL-C), total cholesterol (TC), triglycerides (TG), troponin T (TnT), and D-dimer. Angiographic data and procedure information were extracted from the medical record, including whether stenting, multivessel disease, thrombolysis, and timely PCI were performed. Moreover, we obtained echocardiographic data and information on medications at discharge (calcium channel blockers (CCB), angiotensin II receptor blockers (ARB), angiotensin-converting enzyme inhibitors (ACEI), and beta-blockers. The TyG-BMI index was calculated as ln[TG(mg/dL) × FBG(mg/dL)/2] × BMI.

Patients were considered to have hypertension if they self-reported it, were taking an antihypertensive drug, or had systolic and/or diastolic blood pressure readings above 140/90 mm Hg. Patients were considered to have diabetes mellitus if they self-reported having the disease, were using insulin or oral hypoglycemic medicine, or were confirmed to fit the criteria during an examination after being admitted to the hospital. Age, Killip classification, heart rate, the presence of cardiac arrest during the visit, ST-segment deviation, serum creatinine, systolic blood pressure, and positive cardiac biomarkers were the eight factors used to create a Global Registry of Acute Coronary Events (GRACE) risk score for each patient^24^. Timely PCI was defined as coronary interventions carried out on STEMI patients within 90 min of the initial medical examination^25^. All information was gathered retroactively with the aid of standardized data collection forms. Through outpatient, readmission, or telephone contact, follow-up data was gathered.

### Endpoints

Major adverse cardiovascular events, which include all-cause mortality, target vessel revascularization, nonfatal myocardial infarction, and nonfatal stroke, served as the study's main endpoint. Target vessel revascularization, nonfatal myocardial infarction, nonfatal stroke, and all-cause mortality were secondary endpoints. Fatalities from any cause throughout the follow-up period, including cardiac and noncardiac fatalities, were referred to as all-cause deaths.

### Statistical analysis

The TyG-BMI index tertiles and the incidence of MACE during follow-up were used to divide patients. The continuous variables were described as mean ± standard deviation, and median (interquartile interval). Counts and percentages are utilized to express categorical variables. The chi-square test, or Fisher's exact test, was used to compare clinical data between groups for categorical variables, while the ANOVA, or Kruskal–Wallis test, was used for continuous data. Using either the Pearson or Spearman correlation analysis, it was assessed if the TyG-BMI index was related to cardiovascular risk variables.

Kaplan–Meier curves were used to represent the incidence of MACE, and the log-rank test was used to assess the significance of any differences between the various groups. For each covariate, a univariate Cox regression analysis was conducted. We conducted a multivariate Cox proportional risk regression analysis to further assess whether the TyG-BMI index was a reliable predictor of MACE incidence. There were three multivariate Cox regression models: Model 1 was adjusted for gender and age; Model 2 was further adjusted for smoker, drinker, hypertension, diabetes, previous myocardial infarction (MI), previous arrhythmia, previous PCI, previous stroke, family history of coronary artery disease (FH-CAD), thrombolysis, multi-vessel disease, stenting, timely PCI, left ventricle ejection fraction (LVEF), left ventricular end-diastolic dimension (LVEDD); Model 3 was further adjusted from Model 2 with Killip class ≥ 2, anterior MI on ECG , TIMI grade 0–1 fow before PCI , Time from symptom onset to hospital arrival TC, LDL-C, HDL-C, CK, CKMB, TnT, D-dimer, BUN, Ccr, and use of ACEI/ARB, beta-blockers, and CCB drugs. We determined *p* values for interactions and conducted subgroup analyses for sex, age, smoking, alcohol consumption, timely PCI, diabetes, and hypertension. We represented the TyG index's ability to predict MACE using an area under the curve (AUC) placed on the receiver operating characteristic curve (ROC) curve. To ascertain if raising the TyG-BMI index raises the predictive value of MACE, we compared variations in the Net Reclassification Improvement (NRI), Integrated Discriminant Improvement (IDI), and C-statistic between models. R software (version 4.2.2) was utilized to perform all statistical analyses. The two-sided *p* < 0.05 was considered statistically significant.

## Results

### Characteristics of the study population

This study comprised 2648 patients in total, with a mean age of 59.34 ± 11.40 years, of whom 2144 (81.0%) were male. The baseline characteristics of the study cohort, stratified according to the TyG-BMI index, are displayed in Table 1. Patients who exhibited a greater TyG-BMI index tended to be younger, had a history of PCI and alcohol use, and had concomitant hypertension and diabetes mellitus. Furthermore, these individuals had higher levels of FPG, BMI, TG, TC, LDL, D-dimer, LVEDD, and beta-blocker usage (all *p* < 0.05). In Supplementary Table S1, the baseline characteristics of patients with and without MACE were presented. Patients with adverse cardiovascular events exhibited higher BMI, HR, FPG, TG, TC, LDL, D-dimer, LVEF, and LVEDD (all *p* < 0.05) and were more likely to have had myocardial infarction or stroke in the past. Patients in the MACE group had a substantially higher TyG-BMI index than those in the no-event group compared to the control group (*p* < 0.05).

**Table 1:** Baseline characteristics of the study population according to the TyG-BMI index.

Variables	Total (n = 2648)	Q1 (n = 883)	Q2 (n = 882)	Q3 (n = 883)	*p-*value
TyG-BMI	224.01(204.16–247.48)	193.79(180.23–204.12)	224.01(217.82–231.27)	258.00(247.48–277.93)	< 0.001
Age, years	59.34 ± 11.40	62.15 ± 11.50	59.64 ± 10.93	56.24 ± 11.00	< 0.001
Male	2,144 (81.0)	705 (79.8)	719 (81.5)	720 (81.5)	0.580
Smoker	1,633 (61.7)	533 (60.4)	533 (60.4)	567 (64.2)	0.163
Drinker	870 (32.9)	254 (28.8)	293 (33.2)	323 (36.6)	0.002
Hypertension	1,205 (45.5)	328 (37.1)	428 (48.5)	449 (50.8)	< 0.001
Diabetes	565 (21.3)	112 (12.7)	160 (18.1)	293 (33.2)	< 0.001
Previous PCI	263 (9.9)	73 (8.3)	85 (9.6)	105 (11.9)	0.037
Previous MI	360 (13.6)	114 (12.9)	99 (11.2)	147 (16.6)	0.003
Previous stroke	205 (7.7)	76 (8.6)	66 (7.5)	63 (7.1)	0.481
Previous arrhythmia	315 (11.9)	98 (11.1)	104 (11.8)	113 (12.8)	0.541
FH-CAD	164 (6.2)	50 (5.7)	57 (6.5)	57 (6.5)	0.725
BMI, kg/m^2^	25.06 ± 4.45	22.00 ± 2.10	24.62 ± 1.34	28.57 ± 5.60	< 0.001
SBP, mm Hg	127.52 ± 21.63	125.27 ± 20.78	128.66 ± 21.71	128.62 ± 22.22	< 0.001
DBP, mmHg	77.02 ± 13.65	75.30 ± 12.92	77.37 ± 13.60	78.40 ± 14.23	< 0.001
HR, bpm	75.68 ± 16.90	74.36 ± 16.21	76.28 ± 17.35	76.42 ± 17.06	0.001
GRACE score	142(124–162)	149(130–167)	143.50(126–162)	134(119–156)	< 0.001
FPG, mmol/L	7.85 ± 3.44	6.75 ± 2.35	7.86 ± 3.44	8.94 ± 3.97	< 0.001
BUN, mmol/L	6.07 ± 2.44	6.25 ± 2.94	5.89 ± 1.93	6.07 ± 2.33	0.010
Ccr, ml/min	86.02 ± 24.04	86.82 ± 25.12	85.48 ± 24.05	85.75 ± 22.89	0.213
TG, mmol/L	1.54(1.09–2.25)	1.10(0.82–1.51)	1.60(1.20–2.14)	2.17(1.53–3.07)	< 0.001
TC, mmol/L	4.50(3.81–5.27)	4.30(3.62–4.97)	4.54(3.85–5.33)	4.70(3.99–5.43)	< 0.001
LDL-C, mmol/L	2.42(2.02–2.91)	2.32(1.96–2.77)	2.45(2.03–2.93)	2.48(2.11–3.00)	< 0.001
HDL-C, mmol/L	1.29(1.02–1.57)	1.24(1.01–1.48)	1.31(1.03–1.60)	1.32(1.03–1.62)	< 0.001
D-dimer, ng/ml	0.39 ± 0.34	0.38 ± 0.34	0.40 ± 0.37	0.40 ± 0.30	0.007
TnT, ng/ml	0.21(0.05–0.95)	0.20(0.05–1.04)	0.24(0.05–1.00)	0.18(0.05–0.75)	0.051
CK, U/L	165.00(78.75–615.25)	160.00(74.50–578.50)	187.00(85.00–720.75)	155.00(77.00–531.50)	0.009
CK-MB, U/L	19.00(11.00–56.00)	19.00(12.00–54.00)	21.00(12.00–68.50)	17.00(11.00–49.00)	0.008
LVEF	0.57 ± 0.10	0.57 ± 0.10	0.56 ± 0.09	0.58 ± 0.09	0.003
LVEDD, mm	48.65 ± 5.85	48.14 ± 6.14	48.61 ± 5.61	49.20 ± 5.74	< 0.001
Killip class ≥ 2	571(21.6)	177 (20.0%)	212 (24.0%)	182 (20.6%)	0.088
Anterior MI on ECG	1,427(53.9%)	490(55.5%)	484(54.9%)	453(51.3%)	0.162
TIMI grade 0–1 fow before PCI	2,494(94.2%)	833(94.3%)	830(94.1%)	831(94.1%)	0.972
Time from symptom onset to hospital arrival, min	126.47 ± 146.23	125.20 ± 137.75	115.92 ± 122.42	138.28 ± 173.15	0.045
Stenting	2,591 (97.8)	868 (98.3)	867 (98.3)	856 (96.9)	0.076
Multi-vessel disease	1,773 (67.0)	592 (67.0)	598 (67.8)	583 (66.0)	0.700
Thrombolysis	414 (15.6)	132 (14.9)	146 (16.6)	136 (15.4)	0.600
Timely PCI	878 (33.2)	272 (30.8)	318 (36.1)	288 (32.6)	0.059
Length of stay, days	8.71 ± 10.47	8.66 ± 10.46	8.39 ± 5.10	9.08 ± 13.90	0.900
Medical treatment					
ACEI/ARB	1,220(46.1)	390 (44.2)	422 (47.8)	408(46.2)	0.299
Beta-blockers	1,401 (52.9)	438 (49.6)	468(53.1)	495 (56.1)	0.025
CCB	152 (5.7)	56 (6.3)	63 (7.1)	33 (3.7)	0.006

Data were expressed as mean ± SD, median (with interquartile range), or n (%).

*TyG-BMI* Triglyceride glucose-body mass index, *PCI* percutaneous coronary intervention, *MI* myocardial infarction, *FH-CAD* family history of coronary artery disease, *BMI* body mass index, *SBP* systolic blood pressure, *DBP* diastolic blood pressure, *HR* heart rate, *GRACE Score* Global Registry of Acute Coronary Events Score, *FPG* Fasting plasma glucose, *BUN* Blood urea nitrogen, *Ccr* creatinine clearance rate, *TG* triglyceride, *TC* total cholesterol, *LDL-C* low density lipoprotein cholesterol, *HDL-C* high density lipoprotein cholesterol, *TnT Troponin T, CK* creatine kinase, *CK-MB* creatine kinase-MB, *LVEF* left ventricle ejection fraction, *LVEDD* left ventricular end-diastolic dimension, *ACEI* angiotensin II coenzyme inhibitor, *ARB* angiotensin II receptor blocker, *CCB* calcium channel blocker.

### Correlations between the TyG-BMI index and cardiovascular risk factors

According to the results shown in Table 2, the TyG-BMI index had a positive correlation with TG, TC, LDL-C, BMI, SBP, DBP, FPG, D-dimer, LVEF, and LVEDD and a negative correlation with age (all *p* < 0.05). There was no significant correlation observed between the TyG-BMI index and CCR, TnT, and CK.

**Table 2:** The correlation between TyG-BMI index and baseline clinical risk factors.

Variables	Correlation coefficient (r)	*p*-value
Age, years	− 0.227	< 0.001
LVEF	0.044	0.022
LVEDD, mm	0.100	< 0.001
HDL-C, mmol/L	0.083	< 0.001
LDL-C, mmol/L	0.128	< 0.001
TC, mmol/L	0.169	< 0.001
TG, mmol/L	0.564	< 0.001
BMI, kg/m^2^	0.859	< 0.001
Ccr, ml/min	− 0.032	0.099
SBP, mm Hg	0.096	< 0.001
DBP, mmHg	0.095	< 0.001
FPG, mmol/L	0.299	< 0.001
TnT, ng/ml	− 0.032	0.093
CK, U/L	− 0.005	0.796
D-dimer, ng/ml	0.063	0.001

*LVEF* left ventricle ejection fraction, *LVEDD* left ventricular end-diastolic dimension, *HDL-C* high density lipoprotein cholesterol, *LDL-C* low density lipoprotein cholesterol, *TC* total cholesterol, *TG* triglyceride, *BMI* body mass index, *Ccr* creatinine clearance rate, *SBP* systolic blood pressure, *DBP* diastolic blood pressure, *FPG* Fasting plasma glucose, *TnT Troponin T, CK* creatine kinase.

### The TyG-BMI index and cardiovascular incidents

**Figure 2:**
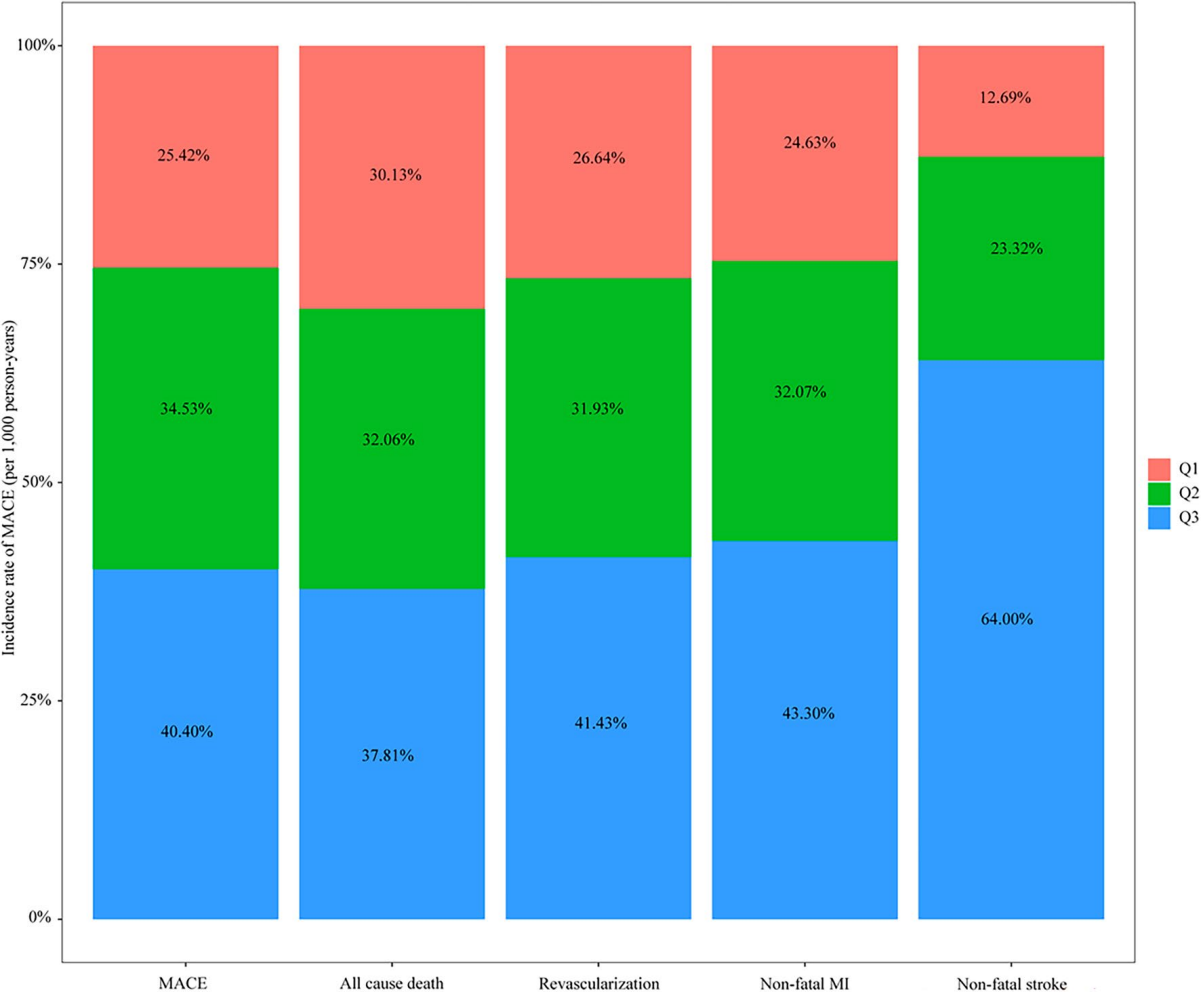
Incidence rates per 1000 person-years of MACE, all-cause death, revascularization, non-fatal myocardial infarction, and non-fatal stroke in the study population by the TyG-BMI index.

**Figure 3:**
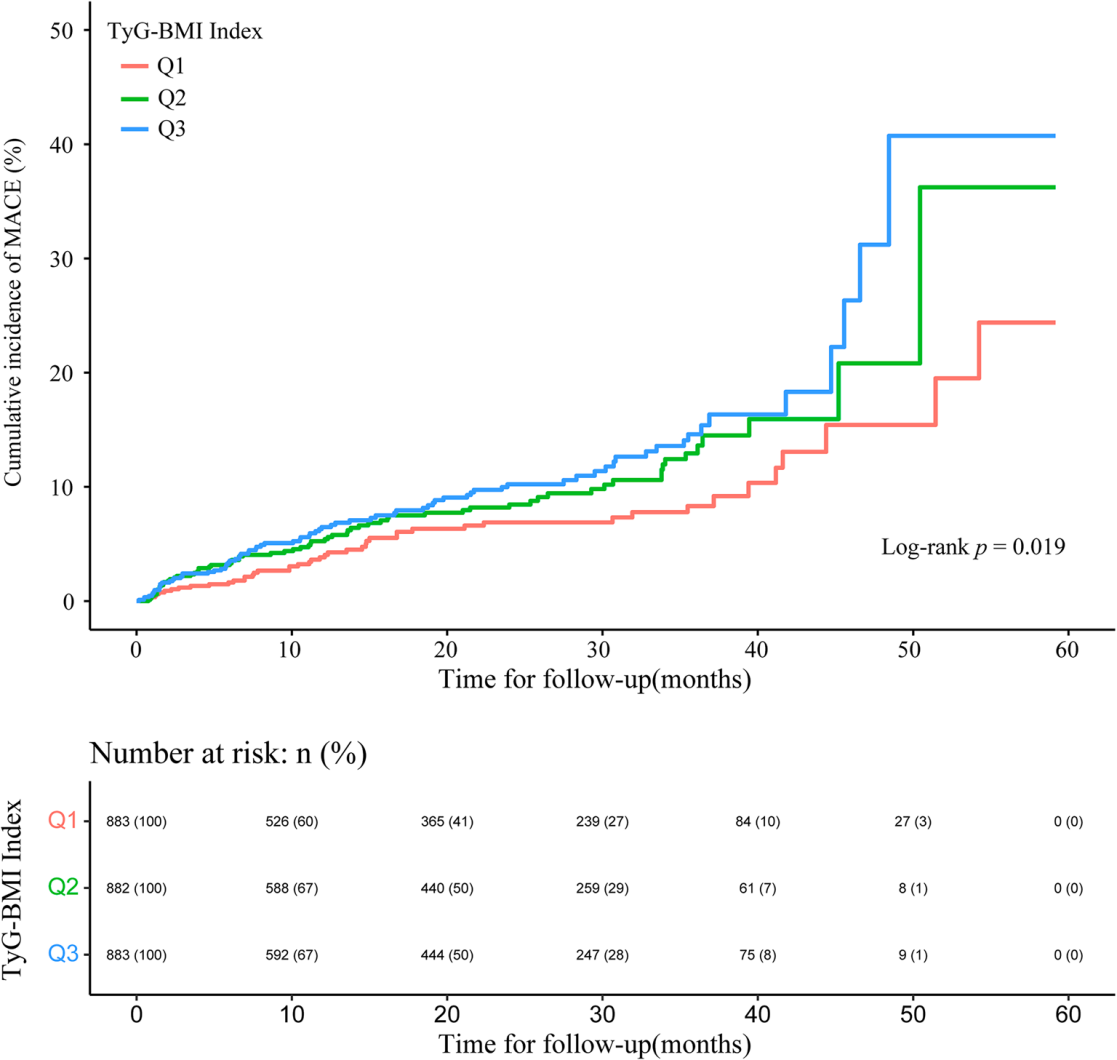
Kaplan–Meier survival curve for MACE by the TyG-BMI index.

In this study, the average follow-up was 14.70 (6.00–32.43) months. Table 3 summarizes the incidence of MACE and specific incidents. A total of 193 patients (7.3%) experienced at least one MACE throughout the follow-up period. With rising the TyG-BMI index, the risk of compound MACE and nonfatal stroke rose (both *p* < 0.05). There were no significant differences in all-cause mortality, revascularization rate, or nonfatal myocardial infarction rate across the three groups. The incidence of composite MACE and individual events per 1000 person-years is illustrated in Fig. 2. For the cumulative incidence of MACE events, we constructed Kaplan–Meier survival curves (Fig. 3). With a higher TyG-BMI index, the cumulative incidence of MACE tended to increase (log-rank *p* = 0.019).

**Table 3:** Adverse cardiovascular events in the study population during follow-up.

	Total (n = 2648)	Q1 (n = 883)	Q2 (n = 882)	Q3 (n = 883)	*p*-value
MACE, (%)	193 (7.3)	46 (5.2)	68 (7.7)	79 (8.9)	0.009
All cause death, (%)	67 (2.5)	19 (2.2)	22 (2.5)	26 (2.9)	0.568
Revascularization, (%)	92 (3.5)	23 (2.6)	30 (3.4)	39 (4.4)	0.114
Non-fatal MI, (%)	52 (2.0)	12 (1.4)	17 (1.9)	23 (2.6)	0.168
Non-fatal stroke, n (%)	17 (0.6)	2 (0.2)	4 (0.5)	11 (1.2)	0.019

*MACE* Major adverse cardiovascular events, *MI* myocardial infarction.

To identify characteristics connected to MACE, we utilized univariate Cox regression analysis (Supplementary Table S2). Body mass index, high blood pressure, diabetes, prior strokes, LVEF, LVEDD, BUN, LDL-C, CKMB, FPG, CCB usage, and the TyG-BMI index were discovered to be risk factors for MACE. After controlling for covariates, multivariate Cox proportional risk regression analysis revealed that the TyG-BMI index was still strongly linked with MACE occurrences (Table 4). The adjusted HRs for the incidence of MACE events in the middle and highest quartiles of the TyG-BMI index compared with the lowest quartile were 1.37 (95% CI 0.92, 2.03) and 1.53 (95% CI 1.02, 2.29), respectively, in the fully adjusted model. With the TyG-BMI index rising, the incidence of MACE rose statistically significantly (*p* for trend = 0.038). We also investigated the relationships between the TyG-BMI index and coronary revascularization, nonfatal myocardial infarction, nonfatal stroke, and overall mortality. The TyG-BMI index was discovered to represent an independent risk factor for nonfatal stroke.

**Table 4:** Multivariate Cox regression analyses for MACE in the study population.

	HR (95% CI)			
Model	Q1	Q2	Q3	*p* for trend
MACE				
Numbers of events/total	46/883	68/882	79/883	
Model 1	1 [Reference]	1.48(1.01, 2.16) *	1.75(1.20, 2.56) *	0.004
Model 2	1 [Reference]	1.42(0.97, 2.09)	1.56(1.05, 2.31) *	0.026
Model 3	1 [Reference]	1.37(0.92, 2.03)	1.53(1.02, 2.29) *	0.038
All cause death				
Numbers of events/total	19/883	22/882	26/883	
Model 1	1 [Reference]	1.34(0.72, 2.52)	1.93(1.03, 3.62) *	0.039
Model 2	1 [Reference]	1.19(0.62, 2.26)	1.48(0.76, 2.87)	0.249
Model 3	1 [Reference]	1.02(0.52, 2.01)	1.49(0.75, 2.95)	0.258
Revascularization				
Numbers of events/total	23/883	30/882	39/883	
Model 1	1 [Reference]	1.24(0.72, 2.14)	1.57(0.92, 2.67)	0.097
Model 2	1 [Reference]	1.23(0.70, 2.14)	1.54(0.89, 2.69)	0.126
Model 3	1 [Reference]	1.24(0.70, 2.18)	1.59(0.90, 2.82)	0.109
Non-fatal MI				
Numbers of events/total	12/883	17/882	23/883	
Model 1	1 [Reference]	1.61(0.75, 3.45)	2.44(1.16, 5.15) *	0.019
Model 2	1 [Reference]	1.55(0.72, 3.33)	2.34(1.08, 5.08) *	0.031
Model 3	1 [Reference]	1.21(0.54, 2.71)	2.25(1.00, 5.08)	0.050
Non-fatal stroke				
Numbers of events/total	2/883	4/882	11/883	
Model 1	1 [Reference]	2.59(0.45, 15.0)	6.82(1.36, 34.3) *	0.020
Model 2	1 [Reference]	2.62(0.42, 16.2)	5.73(1.00, 32.8)	0.050
Model 3	1 [Reference]	2.71(0.38,19.0)	7.20(1.11, 46.5) *	0.038

Model 1: adjusted for age and gender; Model 2: further adjusted (from Model 1) for smoker, drinker, Hypertension, Diabetes, Previous MI, Previous PCI, Previous stroke, Previous arrhythmia, FH-CAD, Thrombolysis, Multi-vessel disease, Stenting, Timely PCI, LVEF, LVEDD; Model 3: further adjusted (from Model 2) for Killip class ≥ 2, anterior MI on ECG , TIMI grade 0–1 fow before PCI , Time from symptom onset to hospital arrival, TC, LDL-C, HDL-C, CK, CKMB, TnT, D-dimer, BUN, Ccr, use of ACEI/ARB, Beta-blockers, and CCB drugs. *MACE* major adverse cardiovascular events, *HR* hazard ratio, *CI* confidence interval, *PCI* percutaneous coronary intervention, *MI* myocardial infarction, *FH-CAD* family history of coronary artery disease, *HR* heart rate, *BUN* Blood urea nitrogen, *Ccr* creatinine clearance rate, *TG* triglyceride, *TC* total cholesterol, *LDL-C* low density lipoprotein cholesterol, *HDL-C* high density lipoprotein cholesterol, *TnT Troponin T, CK* creatine kinase, *CK-MB* creatine kinase-MB, *LVEF* left ventricle ejection fraction, *LVEDD* left ventricular end-diastolic dimension, *ACEI* angiotensin II coenzyme inhibitor, *ARB* angiotensin II receptor blocker, *CCB* calcium channel blocker, *TIMI* Thrombolysis In Myocardial Infarction.

*p < 0.05.

### Subgroup analysis

**Figure 4:**
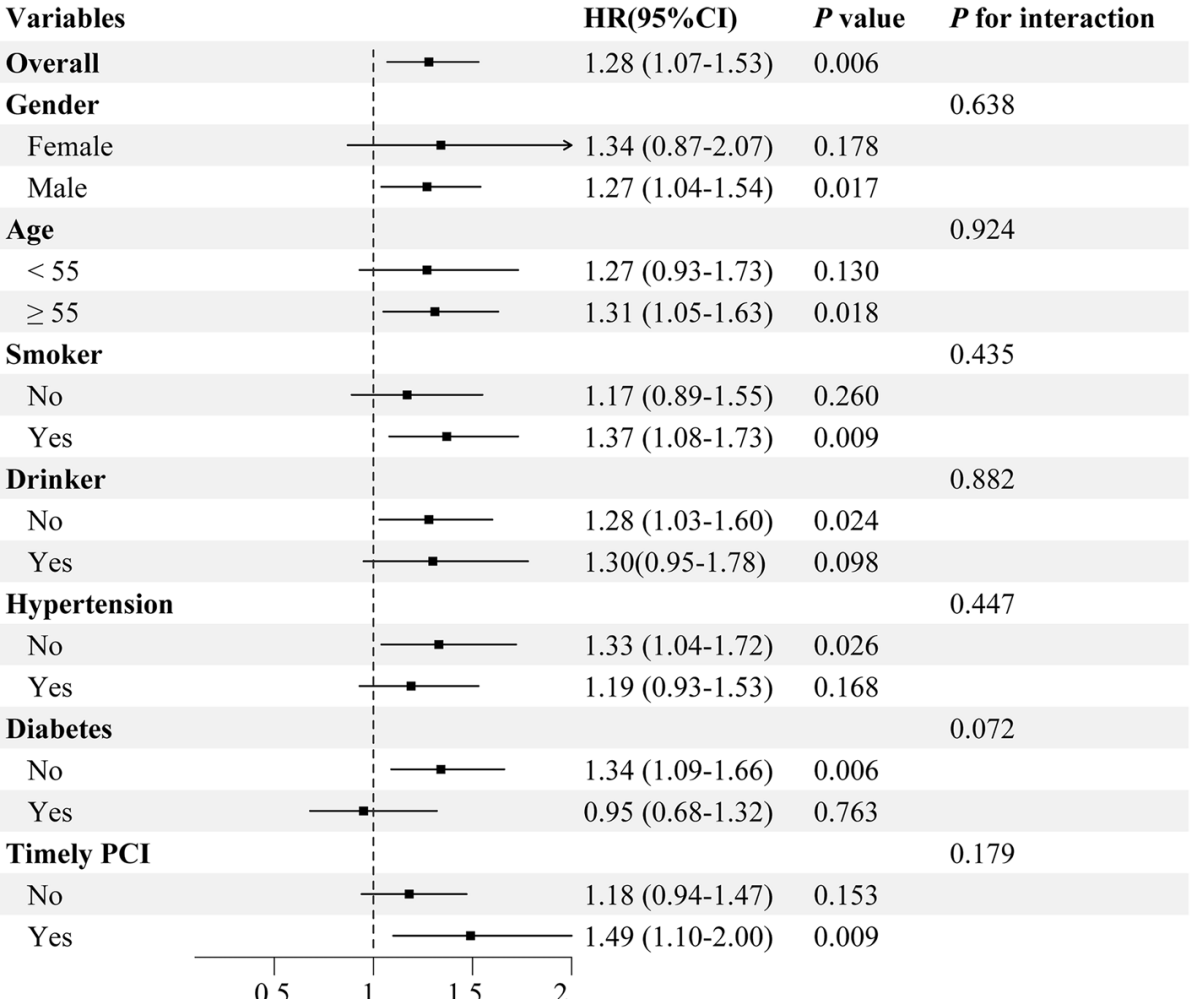
Subgroup analysis between MACE and the TyG-BMI index (Per SD) in various subgroups.

The value of the TyG-BMI index in predicting adverse cardiovascular events was further evaluated in different subgroups of the study population, including gender, age, smoking, alcohol consumption, timely PCI, diabetes, and hypertension (Fig. 4). Differences were statistically significant in the subgroup of patients who were men under 55 years of age, smokers, non-drinkers, non-hypertensive, non-diabetic, and who had timely PCI. Furthermore, no significant interactions between these major stratification factors and the TyG-BMI index were discovered (all *p*_*interaction*_ > 0.05).

### Assessment of the prognostic effect of the TyG-BMI index

**Figure 5:**
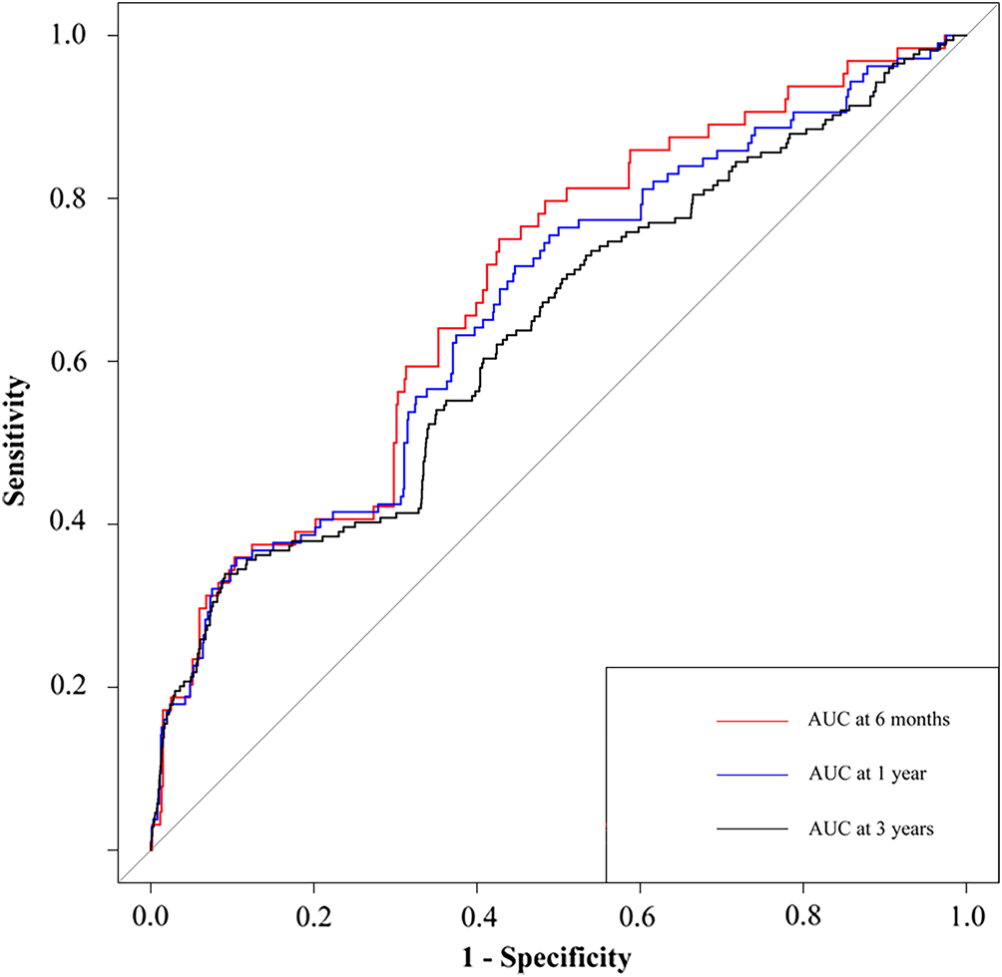
The receiver operating characteristic curves of the TyG-BMI index to predict MACE.

To further evaluate the TyG-BMI index's prognostic significance and predictive capability, we performed a ROC analysis, and the area under the curve at six months, one year, and three years achieved values of 0.691 (95% CI 0.622,0.759), 0.666 (95% CI 0.609,0.723), and 0.637 (95% CI 0.591,0.683), respectively (Fig. 5). Table 5 presents the TyG-BMI incremental predictive values for MACE. The TyG-BMI index dramatically improved the risk classification for MACE by significantly raising the C-statistic, NRI, and IDI (all *p* < 0.05).

**Table 5:** The incremental predictive value of the TyG-BMI index for MACE.

	C-Statistic	*p*-value	NRI (95% CI)	*p*-value	IDI (95% CI)	*p*-value
6 Months MACE						
Model 3 without TyG-BMI	0.791(0.718,0.864)	Reference	Reference	Reference	Reference	Reference
Model 3	0.797(0.724,0.870)	0.001	0.455(0.200,0.710)	< 0.001	0.038(0.013,0.063)	0.003
1 Year MACE						
Model 3 without TyG-BMI	0.791(0.744,0.838)	Reference	Reference	Reference	Reference	Reference
Model 3	0.807(0.763,0.849)	0.006	0.333(0.134,0.532)	0.001	0.034(0.013,0.055)	0.002
3 Years MACE						
Model 3 without TyG-BMI	0.648(0.605,0.691)	Reference	Reference	Reference	Reference	Reference
Model 3	0.681(0.634,0.728)	< 0.001	0.257(0.104,0.409)	0.001	0.040(0.022,0.058)	< 0.001

Model 3: adjusted for age, gender, smoker, drinker, Hypertension, Diabetes, Previous MI, Previous PCI, Previous stroke, Previous arrhythmia, FH-CAD, Thrombolysis, Multi-vessel disease, Stenting, Timely PCI, LVEF, LVEDD, Killip class ≥ 2, anterior MI on ECG , TIMI grade 0–1 fow before PCI , Time from symptom onset to hospital arrival, TC, LDL-C, HDL-C, CK, CKMB, TnT, D-dimer, BUN, Ccr, use of ACEI/ARB, Beta-blockers, and CCB drugs. *MACE* major adverse cardiovascular events, *HR* hazard ratio, *CI* confidence interval, *PCI* percutaneous coronary intervention, *MI* myocardial infarction, *FH-CAD* family history of coronary artery disease, *HR* heart rate, *BUN* Blood urea nitrogen, *Ccr* creatinine clearance rate, *TG* triglyceride, *TC* total cholesterol, *LDL-C* low density lipoprotein cholesterol, *HDL-C* high density lipoprotein cholesterol, *TnT Troponin T, CK* creatine kinase, *CK-MB* creatine kinase-MB, *LVEF* left ventricle ejection fraction, *LVEDD* left ventricular end-diastolic dimension, *ACEI* angiotensin II coenzyme inhibitor, *ARB* angiotensin II receptor blocker, *CCB* calcium channel blocker, *TIMI* Thrombolysis In Myocardial Infarction.

## Discussion

This study is the first that we are aware of that looks at the connection between the TyG-BMI index and MACE events in STEMI patients undergoing PCI. The following was the study's main findings: (1) Independent of conventional cardiovascular risk variables, the TyG-BMI index was associated with the incidence of MACE in STEMI patients. (2) The majority of individuals who had an association between the TyG-BMI index and MACE were male, under the age of 55, smokers, abstainers from alcohol, non-hypertensives, and non-diabetics. (3) The TyG-BMI index's incorporation into prediction models improved prognostic forecasting in STEMI patients. In conclusion, our research demonstrated the TyG-BMI index's predictive significance for MACE in STEMI patients.

The risk of MACE and all-cause mortality is still high in patients with STEMI, despite considerable advancements in PCI therapy over the last few decades, which have resulted in a large drop in mortality. However, prior research has concentrated on conventional cardiovascular risk variables, leaving a gap in the optimization of risk categorization for STEMI patients^26^. Metabolic syndrome, dyslipidemia, obesity, and type 2 diabetes have all been linked to insulin resistance as a significant risk factor^27–29^. Impaired insulin sensitivity is regarded as a key factor in the development of disorders linked to atherosclerosis, such as oxidative stress, endothelial dysfunction, inflammation, metabolic abnormalities, and hypertension^30–32^. Earlier research has demonstrated that IR is a significant risk factor for cardiovascular disease^33^. Obesity has a substantial correlation with IR, and the TyG index is a valid indicator of it. The TyG-BMI index has been demonstrated to be superior to other IR parameters evaluated by HOMA-IR in recent investigations^19^. As a simple replacement for IR, the TyG-BMI index has been proven to have predictive significance in coronary heart disease^34^. The TyG-BMI index's predictive significance in patients with STEMI, however, remained unknown.

Our findings in the current study demonstrated a relationship between the TyG-BMI index and other risk variables. The TyG-BMI index has been linked to prehypertension and hypertension in other research^35,36^, which is congruent with our findings. The association between the TyG-BMI index and the prognosis of STEMI patients with PCI was also revealed for the first time, which is more significant. We discovered a positive relationship between the TyG-BMI index and MACE in the study's participant group. Even though we made adjustments for all study-related risk variables, the outcome remained unchanged. However, recent research that included 2533 people who had drug-eluting stent implantation and percutaneous coronary intervention at the same time revealed no correlation between TyG-BMI and MACE^37^. Differences in the research populations and the incidence of MACE may account for this variance. According to data from a different study of 1092 acute coronary syndrome patients who underwent PCI^7^, greater TyG index values are associated with a higher risk of MACE in patients with STEMI, and the TyG index might be a reliable indicator of clinical outcomes in STEMI patients who had PCI. In terms of IR prediction, the TyG-BMI index is superior to the TyG index^19^. We demonstrated that in STEMI patients undergoing PCI, the TyG-BMI index may be a reliable predictor of MACE. By our findings, the TyG-BMI index has also been demonstrated to have an independent linear connection with ischemic stroke without a threshold or saturation effect^38^. The prediction of MACE in patients following PCI and patients with early-onset coronary artery disease was improved by the addition of the TyG index to baseline risk models, according to prior research^39^. According to our study, the TyG-BMI index added considerable additive prognostic value to predicting MACE in STEMI patients undergoing PCI.

Although the precise molecular processes behind the relationship between the TyG-BMI index and MACE are unclear, important pathways may be connected to IR in STEMI patients undergoing PCI. The traditional CVD risk factors of lipids, glucose, and obesity are included in the TyG-BMI index, which is an accurate predictor of IR. Insulin resistance tended to cause a variety of metabolic disorders, such as hyperglycemia, dyslipidemia, and hypertension, which were closely correlated with a poor prognosis of cardiovascular disease. Insulin resistance-related glycemic and dyslipidemic abnormalities can inhibit nitric oxide production, produce excessive reactive oxidative stress, and cause the deposition of matrix proteins and fibrosis, all of which exacerbate the inflammatory response, encourage the formation of foam cells, impair endothelial function, and encourage the proliferation of smooth muscle cells^8,40^. By raising levels of tissue factor and fibrinogen activator inhibition, IR can also reduce fibrinolysis. This may enhance thrombosis in the cardiovascular system and encourage platelet aggregation^41^. Furthermore, IR increases excessive glycosylation, which promotes the proliferation of vascular smooth muscle cells as well as cross-linking and deposition of collagen, all of which contribute to cardiac fibrosis and stiffening of the diastolic left ventricle^42^. Finally, IR-induced ectopic angiotensinogen production and incorrect renin–angiotensin–aldosterone system activation result in fluid retention and hypertension, which in turn cause cardiovascular events^43^.

A large number of STEMI patients undergoing PCI were included in this research. As far as we are aware, this is the first study to look at the impact of the TyG-BMI index on the prevalence of MACE in patients with STEMI. In STEMI patients undergoing PCI, this study's findings implied that the TyG-BMI index may be a reliable indicator of clinical outcomes. This research did have certain restrictions, though. First of all, because this investigation was a single-center retrospective analysis, it is challenging to rule out the influence of some residual and unmeasured confounders, particularly the management of metabolic syndrome^44^, dietary practices, and physical activity. The study was limited in its relevance to other populations because it was restricted to Chinese patients. Therefore, more research is required to confirm these findings. Third, we only recorded baseline values for FPG, BMI, and triglyceride levels. The follow-up might have affected these indexes. It is unclear, therefore, whether variations in the TyG-BMI index can forecast cardiovascular outcomes. Fourth, it was unable to determine HOMA-IR values since the majority of the patients in this trial did not have their insulin levels assessed. Further research is required to evaluate the TyG-BMI index's predictive value with HOMA-IR since we were unable to compare the values of the two indices. Fifth, the TyG-BMI index requires access to the patient's FPG, BMI, and triglycerides, and the absence of one is not sufficient to predict MACE. Last but not least, patients' laboratory indices were only evaluated once, which might have resulted in bias owing to measurement error. Additional multicenter, large, prospective investigations may support our findings.

## Conclusion

In conclusion, STEMI patients undergoing PCI who had a high TyG-BMI index had an elevated risk of MACE. TyG-BMI index inclusion in the model exhibited an additional predictive value for MACE prediction. As a result, the TyG-BMI index may be a simple and reliable way to assess MACE risk and prognosis.

### Supplementary Information

Supplementary Tables.

## Data Availability

The datasets used for the present analysis may be made available upon reasonable request by contacting the corresponding author.

## Abbreviations

MACE: Major adverse cardiovascular events
TyG-BMI: Triglyceride glucose-body mass index
PCI: Percutaneous coronary intervention
MI: Myocardial infarction
FH-CAD: Family history of coronary artery disease
BMI: Body mass index
SBP: Systolic blood pressure
DBP: Diastolic blood pressure
HR: Heart rate
GRACE Score: Global Registry of Acute Coronary Events Score
FPG: Fasting plasma glucose
BUN: Blood urea nitrogen
Ccr: Creatinine clearance rate
TC: Total cholesterol
TG: Triglyceride
LDL-C: Low density lipoprotein cholesterol
HDL-C: High density lipoprotein cholesterol
TnT: Troponin T
CK: Creatine kinase
CK-MB: Creatine kinase-MB
LVEF: Left ventricle ejection fraction,
LVEDD: Left ventricular end-diastolic dimension,
CAD: Coronary artery disease
IR: Insulin resistance
HR: Hazard ratios
CI: Confidence interval
KM: Kaplan–Meier
TyG index: Triglyceride glucose index
ROC: Receiver operating characteristic
NRI: Net reclassification improvement
IDI: Integrated discrimination improvement
HOMA-IR: Homeostatic Model Assessment to evaluate IR
STEMI: ST-segment myocardial infarction
ACEI: Angiotensin II coenzyme inhibitors
ARB: Angiotensin II receptor blockers
CCB: Calcium channel blockers
