# Supplementary material for: Triglyceride-glucose body mass index predicts prognosis in patients with ST-elevation myocardial infarction
Source: Sci Rep. 2024 Jan 10;14:976. doi: 10.1038/s41598-023-51136-7 (PMC10782013; doi:10.1038/s41598-023-51136-7)
Supplement: Supplementary file 1 — Supplementary Tables. [file 41598_2023_51136_MOESM1_ESM.docx]

**Table S1** Baseline characteristics of the study population by MACE.

| **Variables** | **Total**  **(n=2,648)** | **Non-MACE group (n=2,455)** | **MACE group**  **(n=193)** | ***P*-value** |
| --- | --- | --- | --- | --- |
| TyG-BMI | 224.01(204.16-247.48) | 223.11(202.99-246.19) | 237.15(216.08-279.85) | <0.001 |
| Age, years | 59.34±11.40 | 59.33±11.33 | 59.49±12.25 | 0.906 |
| Male | 2,144.0 (81.0) | 1,984.0 (80.8) | 160.0 (82.9) | 0.477 |
| Smoker | 1,633.0 (61.7) | 1,519.0 (61.9) | 114.0 (59.1) | 0.440 |
| Drinker | 870.0 (32.9) | 805.0 (32.8) | 65.0 (33.7) | 0.800 |
| Hypertension | 1,205.0 (45.5) | 1,103.0 (44.9) | 102.0 (52.8) | 0.033 |
| Diabetes | 565.0 (21.3) | 508.0 (20.7) | 57.0 (29.5) | 0.004 |
| Previous PCI | 263.0 (9.9) | 238.0 (9.7) | 25.0 (13.0) | 0.145 |
| Previous MI | 360.0 (13.6) | 323.0 (13.2) | 37.0 (19.2) | 0.019 |
| Previous stroke | 205.0 (7.7) | 179.0 (7.3) | 26.0 (13.5) | 0.002 |
| Previous arrhythmia | 315.0 (11.9) | 287.0 (11.7) | 28.0 (14.5) | 0.244 |
| FH-CAD | 164.0 (6.2) | 149.0 (6.1) | 15.0 (7.8) | 0.345 |
| BMI, kg/m2 | 25.06±4.45 | 24.83±3.89 | 28.11±8.37 | <0.001 |
| SBP, mm Hg | 127.52±21.63 | 127.42±21.37 | 128.69±24.75 | 0.516 |
| DBP, mmHg | 77.02±13.65 | 76.95±13.53 | 77.88±15.10 | 0.582 |
| HR, bpm | 75.68±16.90 | 75.54±16.89 | 77.58±16.93 | 0.041 |
| GRACE score | 142(124-162) | 142(125-162) | 142(121-164) | 0.850 |
| FPG, mmol/L | 7.85±3.44 | 7.79±3.36 | 8.60±4.26 | 0.010 |
| BUN, mmol/L | 6.07±2.44 | 6.03±2.39 | 6.63±2.99 | 0.004 |
| Ccr, ml/min | 86.02±24.04 | 86.32±24.03 | 82.18±23.87 | 0.008 |
| TG, mmol/L | 1.54(1.09-2.25) | 1.54(1.09-2.23) | 1.70(1.12-2.50) | 0.044 |
| TC, mmol/L | 4.50(3.81-5.27) | 4.49(3.79-5.25) | 4.68(3.99-5.39) | 0.005 |
| LDL-C, mmol/L | 2.42(2.02-2.91) | 2.41(2.01-2.91) | 2.52(2.13-2.93) | 0.024 |
| HDL-C, mmol/L | 1.29(1.02-1.57) | 1.28(1.02-1.56) | 1.39(1.16-1.69) | <0.001 |
| D-dimer, ng/ml | 0.39±0.34 | 0.39±0.33 | 0.46±0.44 | 0.009 |
| TnT, ng/ml | 0.21(0.05-0.95) | 0.20(0.05-0.91) | 0.24(0.05-1.40) | 0.191 |
| CK, U/L | 165.00(78.75-615.25) | 163.00(78.00-601.00) | 195.00(85.00-830.00) | 0.078 |
| CK-MB, U/L | 19.00(11.00-56.00) | 19.00(11.00-55.50) | 19.00(12.00-76.00) | 0.358 |
| LVEF | 0.57±0.10 | 0.57±0.10 | 0.55±0.10 | 0.025 |
| LVEDD, mm | 48.65±5.85 | 48.59±5.77 | 49.43±6.72 | 0.044 |
| Killip class ≥ 2 | 571(21.6) | 529(21.5) | 42(21.8) | 0.945 |
| Anterior MI on ECG | 1,427(53.9) | 1,327(54.1) | 100(51.8) | 0.548 |
| TIMI grade 0–1 fow before PCI | 2,494(94.2) | 2,316(94.3) | 178(92.2) | 0.228 |
| Time from symptom onset to hospital arrival, min | 126.47±146.23 | 127.12±148.19 | 118.26±118.63 | 0.426 |
| Stenting | 2,591.0 (97.8) | 2,403.0 (97.9) | 188.0 (97.4) | 0.605 |
| Multi-vessel disease | 1,773.0 (67.0) | 1,643.0 (66.9) | 130.0 (67.4) | 0.902 |
| Thrombolysis | 414.0 (15.6) | 376.0 (15.3) | 38.0 (19.7) | 0.107 |
| Timely PCI | 878.0 (33.2) | 804.0 (32.7) | 74.0 (38.3) | 0.112 |
| Length of stay, days | 8.71±10.47 | 8.47±8.84 | 11.74±22.44 | 0.023 |
| Medical treatment | |  |  |  |
| ACEI/ARB | 1,220.0 (46.1) | 1,126.0 (45.9) | 94.0 (48.7) | 0.446 |
| Beta-blockers | 1,401.0 (52.9) | 1,287.0 (52.4) | 114.0 (59.1) | 0.075 |
| CCB | 152.0 (5.7) | 130.0 (5.3) | 22.0 (11.4) | <0.001 |

Data were expressed as mean ± SD, median (with interquartile range), or n (%)

*MACE Major adverse cardiovascular events, TyG-BMI* Triglyceride glucose-body mass index, *PCI* percutaneous coronary intervention, *MI* myocardial infarction, *FH-CAD* family history of coronary artery disease, *BMI* body mass index, *SBP* systolic blood pressure, *DBP* diastolic blood pressure, *HR* heart rate, *GRACE Score* Global Registry of Acute Coronary Events Score, *FPG* Fasting plasma glucose, *BUN* Blood urea nitrogen, *Ccr* creatinine clearance rate, *TG* triglyceride, *TC* total cholesterol, *LDL-C* low density lipoprotein cholesterol, *HDL-C* high density lipoprotein cholesterol, TnT Troponin T, *CK* creatine kinase, *CK-MB* creatine kinase-MB, *LVEF* left ventricle ejection fraction, *LVEDD* left ventricular end-diastolic dimension, *ACEI* angiotensin II coenzyme inhibitor, *ARB* angiotensin II receptor blocker, *CCB* calcium channel blocker.

**Table S2** Univariate Cox regression analyses for MACE in the study population.

| **Variables** | **HR (95% CI)** | ***P*-value** |
| --- | --- | --- |
| TyG-BMI | 1.28 (1.07-1.53) | 0.006 |
| Male | 1.09 (0.75-1.59) | 0.648 |
| Age, years | 1.00 (0.99-1.01) | 0.955 |
| BMI, kg/m^2^ | 1.08 (1.07-1.10) | <0.001 |
| SBP, mm Hg | 1.00 (1.00-1.01) | 0.445 |
| DBP, mmHg | 1.01 (1.00-1.02) | 0.203 |
| HR, bpm | 1.01 (1.00-1.01) | 0.159 |
| Smoker | 0.86 (0.64-1.14) | 0.287 |
| Drinker | 0.98 (0.73-1.32) | 0.903 |
| Hypertension | 1.33 (1.00-1.77) | 0.047 |
| Diabetes | 1.63 (1.19-2.22) | 0.002 |
| Previous MI | 1.34 (0.93-1.91) | 0.114 |
| Previous PCI | 1.20 (0.79-1.82) | 0.401 |
| Previous stroke | 2.02 (1.33-3.05) | 0.001 |
| Previous arrhythmia | 1.08 (0.72-1.61) | 0.711 |
| FH-CAD | 1.09 (0.65-1.86) | 0.736 |
| Killip class ≥ 2 | 1.02 (0.73-1.44) | 0.906 |
| Anterior MI on ECG | 0.94 (0.71-1.25) | 0.679 |
| TIMI grade 0–1 fow before PCI | 0.82 (0.49-1.40) | 0.473 |
| Time from symptom onset to hospital arrival, min | 1.00 (1.00-1.00) | 0.503 |
| Thrombolysis | 1.15 (0.81-1.64) | 0.444 |
| Multi-vessel disease | 1.03 (0.76-1.39) | 0.865 |
| Stenting | 0.84 (0.34-2.03) | 0.694 |
| Timely PCI | 0.95 (0.71-1.27) | 0.736 |
| LVEF | 0.17 (0.04-0.70) | 0.015 |
| LVEDD, mm | 1.02 (1.00-1.04) | 0.018 |
| Beta-blockers | 1.10(0.82-1.46) | 0.520 |
| CCB | 2.14 (1.37-3.34) | 0.001 |
| ACEI/ARB | 1.14 (0.86-1.52) | 0.351 |
| BUN, mmol/L | 1.09 (1.05-1.13) | <0.001 |
| Ccr, ml/min | 0.99 (0.99-1.00) | 0.062 |
| TC, mmol/L | 1.10 (0.97-1.24) | 0.135 |
| TG, mmol/L | 1.09 (1.00-1.19) | 0.059 |
| LDL-C, mmol/L | 1.28 (1.03-1.58) | 0.025 |
| HDL-C, mmol/L | 1.01 (0.70-1.46) | 0.965 |
| CK, U/L | 1.00 (1.00-1.00) | 0.085 |
| CK-MB, U/L | 1.00 (1.00-1.00) | 0.043 |
| TnT, ng/ml | 1.01 (1.00-1.01) | 0.068 |
| D-dimer, ng/ml | 1.08 (0.74-1.58) | 0.691 |
| FPG, mmol/L | 1.05 (1.01-1.09) | 0.005 |
| GRACE score | 1.00 (1.00-1.01) | 0.701 |

*MACE Major adverse cardiovascular events, TyG-BMI* Triglyceride glucose-body mass index, *PCI* percutaneous coronary intervention, *MI* myocardial infarction, *FH-CAD* family history of coronary artery disease, *BMI* body mass index, *SBP* systolic blood pressure, *DBP* diastolic blood pressure, *HR* heart rate, *GRACE Score* Global Registry of Acute Coronary Events Score, *FPG* Fasting plasma glucose, *BUN* Blood urea nitrogen, *Ccr* creatinine clearance rate, *TG* triglyceride, *TC* total cholesterol, *LDL-C* low density lipoprotein cholesterol, *HDL-C* high density lipoprotein cholesterol, TnT Troponin T, *CK* creatine kinase, *CK-MB* creatine kinase-MB, *LVEF* left ventricle ejection fraction, *LVEDD* left ventricular end-diastolic dimension, *ACEI* angiotensin II coenzyme inhibitor, *ARB* angiotensin II receptor blocker, *CCB* calcium channel blocker, *TIMI* Thrombolysis In Myocardial Infarction, *HR* Hazard ratio, *CI* Confidence interval.
